# Bordetella bronchiseptica promotes adherence, colonization, and cytotoxicity of Streptococcus suis in a porcine precision-cut lung slice model

**DOI:** 10.1080/21505594.2020.1858604

**Published:** 2020-12-29

**Authors:** Désirée Vötsch, Maren Willenborg, Wolfgang Baumgärtner, Manfred Rohde, Peter Valentin-Weigand

**Affiliations:** a Institute for Microbiology, University of Veterinary Medicine Hannover, Hannover, Germany; b Institute for Pathology, University of Veterinary Medicine Hannover, Hannover, Germany; c Central Facility for Microscopy, Helmholtz Center for Infection Research, Braunschweig, Germany

**Keywords:** *Bordetella bronchiseptica*, *Streptococcus suis*, suilysin, respiratory co-infection, porcine precision-cut lung slices, porcine respiratory tract infection, PRDC

## Abstract

*Bordetella (B.) bronchiseptica* and *Streptococcus (S.) suis* are major pathogens in pigs, which are frequently isolated from co-infections in the respiratory tract and contribute to the porcine respiratory disease complex (PRDC). Despite the high impact of co-infections on respiratory diseases of swine (and other hosts), very little is known about pathogen-pathogen-host interactions and the mechanisms of pathogenesis. In the present study, we established a porcine precision-cut lung slice (PCLS) model to analyze the effects of *B. bronchiseptica* infection on adherence, colonization, and cytotoxic effects of *S. suis*. We hypothesized that induction of ciliostasis by a clinical isolate of *B. bronchiseptica* may promote subsequent infection with a virulent *S. suis* serotype 2 strain. To investigate this theory, we monitored the ciliary activity by light microscopy, measured the release of lactate dehydrogenase, and calculated the number of PCLS-associated bacteria. To study the role of the pore-forming toxin suilysin (SLY) in *S. suis*-induced cytotoxicity, we included a SLY-negative isogenic mutant and the complemented mutant strain. Furthermore, we analyzed infected PCLS by histopathology, immunofluorescence microscopy, and field emission scanning electron microscopy. Our results showed that pre-infection with *B. bronchiseptica* promoted adherence, colonization, and, as a consequence of the increased colonization, the cytotoxic effects of *S. suis*, probably by reduction of the ciliary activity. Moreover, cytotoxicity induced by *S. suis* is strictly dependent on the presence of SLY. Though the underlying molecular mechanisms remain to be fully clarified, our results clearly support the hypothesis that *B. bronchiseptica* paves the way for *S. suis* infection.

## Introduction

Respiratory diseases in pigs are often caused by bacterial and viral co-infections. However, very little is known about pathogen-pathogen-host interactions and the mechanisms of pathogenesis. The porcine respiratory disease complex (PRDC) is a paradigm for such infections. It is a major cause of mortality and reduced weight gain in the swine population worldwide and, therefore, a serious economic concern for the pig industry. Presumably, pathogenesis of PRDC is based on synergistic interactions of primary and secondary opportunistic pathogens [1–3]. Primary pathogens, including porcine reproductive and respiratory syndrome virus, swine influenza virus (swIAV), *Mycoplasma hyopneumoniae*, and *Bordetella (B.) bronchiseptica*, can cause an infection by themselves, which is usually mild. However, they can also pave the way for secondary pathogens, such as *Pasteurella multocida, Streptococcus (S.) suis*, and *Glaesserella parasuis*, which are frequent commensals in the porcine respiratory tract. The latter, also known as pathobionts [4], not to state that G. parasuis and S. suis are unable to establish infection without a primary pathogen. While they do complicate infection with other pathogens, they are also capable of causing primary systemic infection [1].

In the present study, we have analyzed interactions of two major bacterial pathogens involved in the PRDC, *B. bronchiseptica* and *S. suis*. The former is a Gram-negative bacterium contributing to several secondary bacterial infections [5–9]. In contrast to the closely related human pathogen *B. pertussis, B. bronchiseptica* has a broad host range, including wild, domestic, and companion animals, as well as humans. Additionally, it plays a role in many respiratory diseases, e.g., atrophic rhinitis in swine and kennel cough in dogs. *B. bronchiseptica* is ubiquitous in pig populations and is frequently isolated in combination with other pathogens from cases of pneumonia [10]. Virulence factors of this pathogen include, e.g., filamentous hemagglutinin, fimbriae, pertactin, and toxins, like the dermonecrotic toxin (DNT) or the tracheal cytotoxin (TCT). Most of these are regulated by a two-component signal transduction system in response to environmental conditions (reviewed in [11]).

*S. suis* is a frequent colonizer of the upper respiratory tract of pigs and considered a pathobiont or secondary pathogen, which can become invasive when the respiratory epithelium has been damaged, e.g., by prior infection with a primary viral or bacterial pathogen [4]. Then, *S. suis* can enter the bloodstream and cause severe systemic diseases, such as meningitis, arthritis, endocarditis, and septicemia [12], resulting in high economic losses for the pig industry. Moreover, as an emerging zoonotic agent, *S. suis* can also cause meningitis and septicemia in humans [13,14]. Most of the virulent strains are positive for suilysin (SLY), a secreted extracellular acting pore-forming protein that contributes to virulence of *S. suis* by damaging epithelial cells and promotion of bacterial adherence and invasion [15–17] (reviewed in [18]).

In our previous studies, we have shown in co-infections of porcine precision-cut lung slices (PCLS) that swIAV can induce ciliostasis, thereby promoting adherence and invasion of *S. suis* [19]. This prompted us to investigate possible effects of other (primary) respiratory pathogens on infection of *S. suis*. Earlier experimental infection studies by Vecht *et al*. revealed that *B. bronchiseptica* could predispose piglets to infection with *S. suis*, resulting in more severe clinical signs and elevated bacterial loads in the tissue [6,7]. However, the mechanisms of how *B. bronchiseptica* led to increased colonization of the respiratory epithelium by *S. suis* are still unclear. Possible mechanisms might be the use of adhesins (e.g., filamentous hemagglutinin) secreted by *B. bronchiseptica* [20] or facilitated attachment to respiratory epithelial cells due to ciliostasis [21–23], probably induced by the TCT [24]. Thus, we assumed that *B. bronchiseptica* might promote *S. suis* infection by a similar effect as swIAV, i.e. by reduction of ciliary activity. In order to investigate this hypothesis, we first established a PCLS model for co-infection with *B. bronchiseptica* and *S. suis*. PCLS have been proven as a suitable model to investigate respiratory infections [25–27]. The main advantage of PCLS is the preservation of the original cellular and structural organization of the lung tissue. Moreover, ciliary beating at the bronchiolar surface can be assessed by light microscopy. Using PCLS, we were able to show that prior infection with *B. bronchiseptica* leads to ciliostasis and enhances subsequent adherence, colonization, and, as a consequence of the higher colonization, the cytotoxicity of *S. suis*.

## Materials and methods

### Porcine precision-cut lung slices

For the preparation of PCLS, lungs from apparently healthy pigs of different ages were obtained from a local slaughterhouse (Leine-Fleisch GmbH, Laatzen, Germany). As previously described [19,25,26], the cranial, middle, and intermediate lung lobes were filled with 1.5% (w/v) low-melting agarose (GERBU, Heidelberg, Germany) in RPMI 1640 medium (Sigma-Aldrich, Taufkirchen, Germany) and kept on ice until agarose became solidified. Afterward, cylindrical pieces of lung tissue with a bronchiole in the middle were punched out with a tissue-coring tool and cut into approximately 300 µm thick slices using a Krumdieck tissue slicer (model MD 4000–01; TSE Systems, Chesterfield, MO, USA). Slices were collected in RPMI 1640 medium (Thermo Fisher Scientific, Waltham, MA, USA) supplemented with antibiotics and antimycotics (1 µg/ml clotrimazole, 10 µg/ml enrofloxacin, 50 µg/ml kanamycin,100 U/ml penicillin, 100 µg/ml streptomycin, 50 µg/ml gentamicin, 2.5 µg/ml amphotericin B) and bubbled for approximately 2 hours (h) with a normoxic gas mixture at 37°C to remove agarose from the slices [28]. Then, PCLS were collected in 24-well plates (Greiner Bio-One, Frickenhausen, Germany) and incubated in RPMI 1640 medium supplemented with antibiotics and antimycotics at 37°C and 5% CO_2_ for one day. The next day, slices with good ciliary activity (at least 80–90%, determined by light microscopy) were chosen for experiments, washed with phosphate-buffered saline (PBS; Sigma-Aldrich), and incubated in RPMI 1640 medium without any antibiotics and antimycotics for one day.

### Bacterial strains

We used a clinical lung isolate of *B. bronchiseptica* (*B. bronchiseptica* 1263/2/18), obtained from a swine herd with unspecific clinical symptoms, such as reduced weight gain and sporadic cases of anemic animals. It was grown on Columbia agar supplemented with 7% (v/v) sheep blood (Oxoid™, Thermo Fisher Scientific) for 48 h at 37°C under aerobic conditions.

For infection experiments, cryo-conserved bacterial stocks were prepared. For this, *B. bronchiseptica* was grown overnight at 37°C under aerobic conditions in Brain Heart Infusion (BHI) broth (Bacto™, Becton Dickinson, Heidelberg, Germany), adjusted to an optical density at 600 nm (OD_600_) of 0.05 in prewarmed BHI broth and further incubated until early exponential growth phase (OD_600_ of 0.6). Bacteria were centrifuged (5000 × *g* for 10 min) and resuspended in BHI broth with 15% (v/v) glycerol. Aliquots were then frozen in liquid nitrogen and transferred to −80°C for long-term storage.

The virulent SLY-positive *S. suis* serotype 2 wild-type strain 10 (*S. suis* 10 wt) was kindly provided by H. Smith (Lelystad, Netherlands) [29]. The isogenic SLY-deficient mutant of *S. suis* 10 (*S. suis* 10∆*sly*) was constructed by insertion of an erythromycin cassette in the *sly* gene [30]. The complemented SLY-mutant strain (*S. suis* 10cS148) was generated by chromosomal complementation of *S. suis* 10∆*sly*, carrying a silent mutation of the *sly* gene to delimit this strain from the wt [15]. All *S. suis* strains were grown on Columbia agar supplemented with 7% (v/v) sheep blood (Oxoid™, Thermo Fisher Scientific) overnight at 37°C under aerobic conditions. Cryo-conserved bacterial stocks were prepared from liquid cultures in Todd-Hewitt Broth (THB; Bacto™, Becton Dickinson) at the late-exponential growth phase (OD_600_ of 1.0), as previously described [19].

### Infection of PCLS with B. bronchiseptica and S. suis

Prior to infection experiments, PCLS were maintained without antibiotics and antimycotics for one day and washed two times with PBS.

To establish the infection with *B. bronchiseptica*, PCLS were infected with low doses of *B. bronchiseptica* (approximately 10^2^–10^4^ CFU/well) in 500 µl RPMI 1640 medium in a 24-well plate for 4 h at 37°C and 5% CO_2_. In order to remove non-adherent bacteria, slices were washed two times with PBS and fresh medium was added for further incubation up to 48 hours post-infection (hpi).

For co-infection experiments, PCLS were pre-infected with approximately 10^4^ CFU/well of *B. bronchiseptica* in 500 µl RPMI 1640 medium in a 24-well plate for 4 h at 37°C and 5% CO_2_ and then washed two times with PBS to remove non-adherent bacteria. Fresh medium was added, and slices were incubated for another 20 h at 37°C and 5% CO_2_. PCLS were again washed two times with PBS and then co-infected with approximately 10^7^ CFU/well of *S. suis* 10 wt, *S. suis* 10∆*sly*, and *S. suis* 10cS148, respectively, in 500 µl RPMI 1640 medium for 4 h at 37°C and 5% CO_2_. Afterward, slices were washed two times with PBS to remove non-adherent streptococci, fresh medium was added, and slices were further incubated at 37°C and 5% CO_2_ up to 48 hpi with *S. suis*.

### Ciliary activity

For infection experiments, we chose slices showing rapidly moving cilia on at least 80% of the whole luminal surface of the bronchiole. Ciliary activity of at least two slices was estimated using light microscopy (Leica DMi1; Leica, Wetzlar, Germany) and the activity determined before infection was set as 100%. Ciliary movement of at least two slices was monitored at different times after infection with *B. bronchiseptica* and *S. suis*, respectively, and the experiment was repeated at least three times.

### Cytotoxicity assay

To determine the extent of cytotoxicity caused by *B. bronchiseptica* and/or *S. suis*, we measured the release of lactate dehydrogenase (LDH) at different times using CytoTox 96® Non-Radioactive Cytotoxicity Assay (Promega, Mannheim, Germany). Supernatants of infected and uninfected PCLS were collected and the cytotoxicity assay was performed as recommended by the manufacturer. LDH release of infected PCLS was normalized to uninfected PCLS and results were expressed as percentage LDH release compared to uninfected PCLS lysed with 10% (v/v) Triton® X 100 (Carl Roth, Karlsruhe, Germany) in RPMI 1640 medium. All experiments were performed in duplicates and repeated at least three times.

### Bacterial adherence and colonization

To quantify PCLS-associated bacteria (surface-adherent and/or intracellular bacteria), PCLS were washed two times with PBS and then homogenized in PBS using Lysing Matrix D (MP Biomedicals, Irvine, CA, USA) and the FastPrep-24™ 5 G Instrument (3 × 30 sec, speed 5; MP Biomedicals). Afterward, bacterial number in the lysate was determined by serial dilution and replicate plating on agar plates. To determine the number of *B. bronchiseptica* in mono-infection experiments, the lysate was plated on Columbia agar supplemented with 7% (v/v) sheep blood (Oxoid™, Thermo Fisher Scientific). To differentiate *S. suis* from *B. bronchiseptica*, we used blood agar plates containing 7% (v/v) bovine blood and Oxoid™ Staph/Strep-selective supplement (Thermo Fisher Scientific; referred to as “Staph/Strep-agar plate”). Both, *B. bronchiseptica* and *S. suis* were incubated for 48 h at 37°C under aerobic conditions. All experiments were performed in duplicates and repeated at least three times.

### Immunofluorescence microscopy

v) formalin, embedded in paraffin blocks and sections of 3–4 μm were prepared. For staining purposes, paraffin sections were deparaffi/v) formalin, embedded in paraffin blocks and sections of 3–4 µm were prepared. For staining purposes, paraffin sections were deparaffinized in histol (Carl Roth), rehydrated in descending series of ethanol (100%, 95%, and 70%), and cooked in sodium-citrate buffer (10 mM, pH 6.0, 10 min) to retrieve the antigens. Nonspecific binding sites were blocked using 5% (v/v) goat serum (Sigma-Aldrich), 0.3% (v/v) Triton® X 100 (Carl Roth) and 0.05% (v/v) Tween® 20 (Carl Roth) in PBS. All antibodies were diluted in 1% (v/v) bovine serum albumin (BSA; Carl Roth) and 0.05% (v/v) Tween® 20 in PBS and were incubated for 1 h at room temperature (RT) or overnight at 4°C. *B. bronchiseptica* was stained using a polyclonal rat anti-*B. bronchiseptica* antiserum (1:500; Davids Biotechnologie GmbH, Regensburg, Germany) and an Alexa Fluor® 488 goat-anti-rat IgG (H + L) antibody (1:1,000; Thermo Fisher Scientific). For detection of streptococci we used a polyclonal rabbit anti-*S. suis* antiserum (1:500) [30] and an Alexa Fluor® 647 donkey-anti-rabbit IgG (H + L) antibody (1:1,000; Thermo Fisher Scientific). To visualize ciliated cells, we applied a Cy3-labeled monoclonal mouse antibody against β-tubulin (1:250; Sigma-Aldrich) and nuclei were stained by 4ʹ, 6-diamidino-2-phenylindole (DAPI, 0.5 µg/ml in PBS; Cell Signaling Technology, Beverly, MA, USA). Finally, the sections were mounted with ProLong® Gold Antifade Reagent (Cell Signaling Technology) and stored at 4°C until examination.

Samples were analyzed using the Nikon Eclipse Ti-S (Nikon, Tokyo, Japan), equipped with the objectives Plan Fluor 10x/0.30 DIC and 60x/0.50–1.25 Oil. Brightness and contrast were adjusted using ImageJ/Fiji 1.52p software (National Institute of Health, USA).

### Histological examination

Hematoxylin and eosin (HE) staining of paraffin sections was performed following standard procedures [31]. For examination of the HE stained PCLS, we used the Zeiss AXIO Imager.M2 (Carl Zeiss, Stockholm, Sweden) equipped with the camera AxioCam MRm Rev.3 FireWire (D) and the objective Plan-Apochromat 63x/1.4 Oil.

### Field emission scanning electron microscopy

Sample preparation and examination were performed as described previously [32] with some modifications. Infected PCLS were fixed with 5% (v/v) formaldehyde and 2% (v/v) glutaraldehyde in HEPES buffer (0.1 M HEPES, 0.01 M CaCl_2_, 0.01 M MgCl_2_, 0.09 M sucrose; pH 6.9) at 7°C until dehydration process. Samples were washed twice with TE buffer (0.01 M Tris, 1 mM EDTA; pH 6.9) and dehydrated with graded series of ethanol (10%, 30%, 50%, 70%, 90%, and 100%) on ice for 15 min for each dilution. Samples in 100% ethanol were allowed to reach RT and then exposed to critical point drying with liquid CO_2_ (Bal-Tec CPD 030, Balzers, Liechtenstein). Subsequently, samples were covered with a gold-palladium film (approximately 10 nm) by sputter coating (Bal-Tec SCD500, Balzers) before examination with the field emission scanning electron microscope (FESEM) Carl Zeiss Merlin™ (Carl Zeiss), equipped with the Everhart Thornley SE-detector and the inlense SE-detector in a 50:50 ratio at an acceleration voltage of 5 kV.

### Immunoblot analysis

Supernatants of infected PCLS were separated electrophoretically using a 5% (v/v) stacking and a 10% (v/v) running sodium dodecyl sulfate polyacrylamide gel (SDS-PAGE) and were transferred to a polyvinylidene fluoride (PVDF) membrane (Amersham Hybond-P, GE Healthcare, Chicago, IL, USA). The membranes were blocked for 1 h at RT with 5% (v/v) skimmed milk powder in Tris-buffered saline (TBS) with 1% (v/v) Tween® 20 (Carl Roth) and incubated with purified polyclonal antiserum raised against SLY [84] (diluted 1:2,500 in 1% (v/v) milk powder in TBS with 1% Tween® 20) overnight at 4°C to detect SLY-expression in the supernatant. Development of the membranes was performed with HRP-linked goat anti-rabbit IgG (Cell Signaling) (diluted 1:5,000 in 1% milk powder in TBS with 1% Tween® 20, incubated for 1 h at RT), SuperSignal™ West Pico PLUS Chemiluminescent Substrate (Thermo Fisher Scientific) and chemiluminescence detection with ChemoCam Imager 3.2 (Intas, Göttingen, Germany).

### Statistical analyses

All experiments were repeated at least three times and the data in the figures are shown as the means ± standard deviation (means ± SD). All statistical analyses were carried out using GraphPad Prism version 8.0.1 for Windows (GraphPad Software, San Diego, CA, USA). Normal (Gaussian) distribution of the data was tested by Shapiro-Wilk test and statistical significance was determined by *t*-test or by one-way ANOVA followed by Tukey, Dunnett, or Sidak *post-hoc* test; *p* < 0.05 was considered significant.

## Results

### Establishment of defined conditions for PCLS infection with B. bronchiseptica

**Figure 1.**
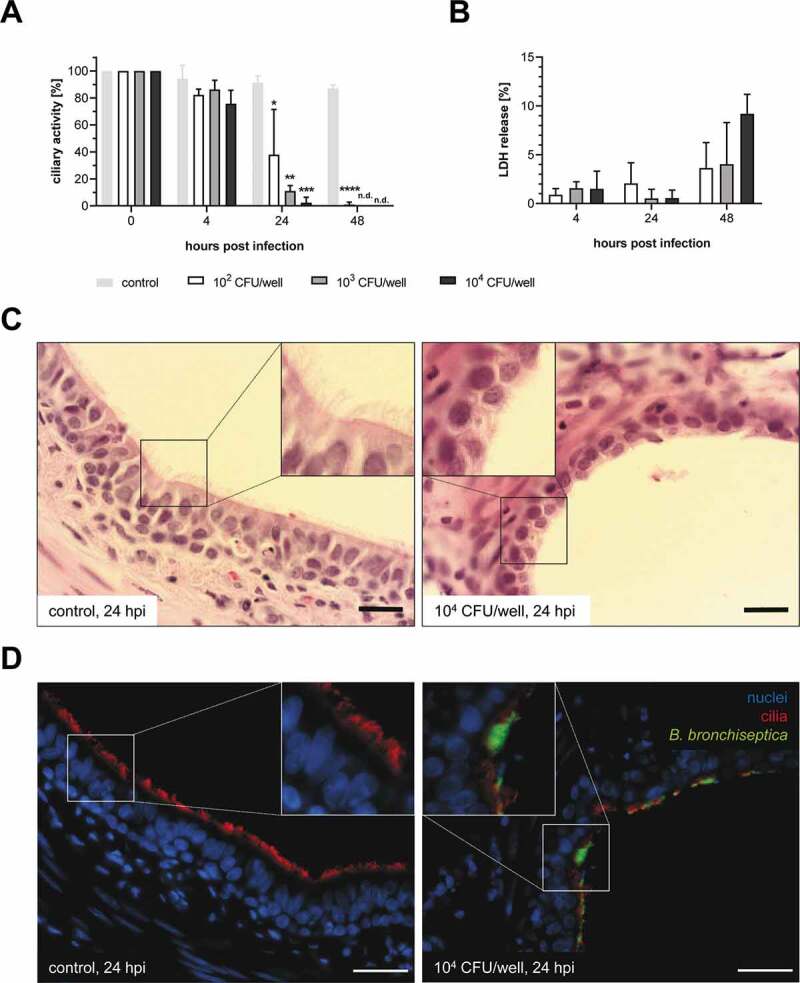
Impact of *B. bronchiseptica* on PCLS. PCLS were infected with 10^2^–10^4^ CFU/well of *B. bronchiseptica* for up to 48 h. (A) Ciliary activity of uninfected (control) and infected PCLS was monitored at indicated time points by estimating the ciliary beating using light microscopy. Results are expressed as percentage ciliary activity compared to the ciliary activity before infection (set as 100%). Significant differences between uninfected and infected PCLS are indicated by * *p* < 0.05, ** *p* < 0.01, *** *p* < 0.001, and **** *p* < 0.0001; one-way ANOVA followed by Dunnett *post-hoc* test. Ciliary activity was not detectable (n.d.). (B) Cytotoxicity was determined by measuring the release of LDH in the supernatant of infected PCLS. Results are expressed as percentage LDH release compared to PCLS lysed with 10% Triton® X 100. Significant differences between the infection doses were analyzed using one-way ANOVA followed by Tukey *post-hoc* test; no significant differences were found. (C) HE staining and (D) immunofluorescence staining of uninfected PCLS and PCLS infected with 10^4^ CFU/well of *B. bronchiseptica* at 24 hpi. Bars represent 20 µm. (D) *B. bronchiseptica* is shown in green, cilia (β-tubulin) in red, and nuclei (DAPI) in blue

**Figure 2.**
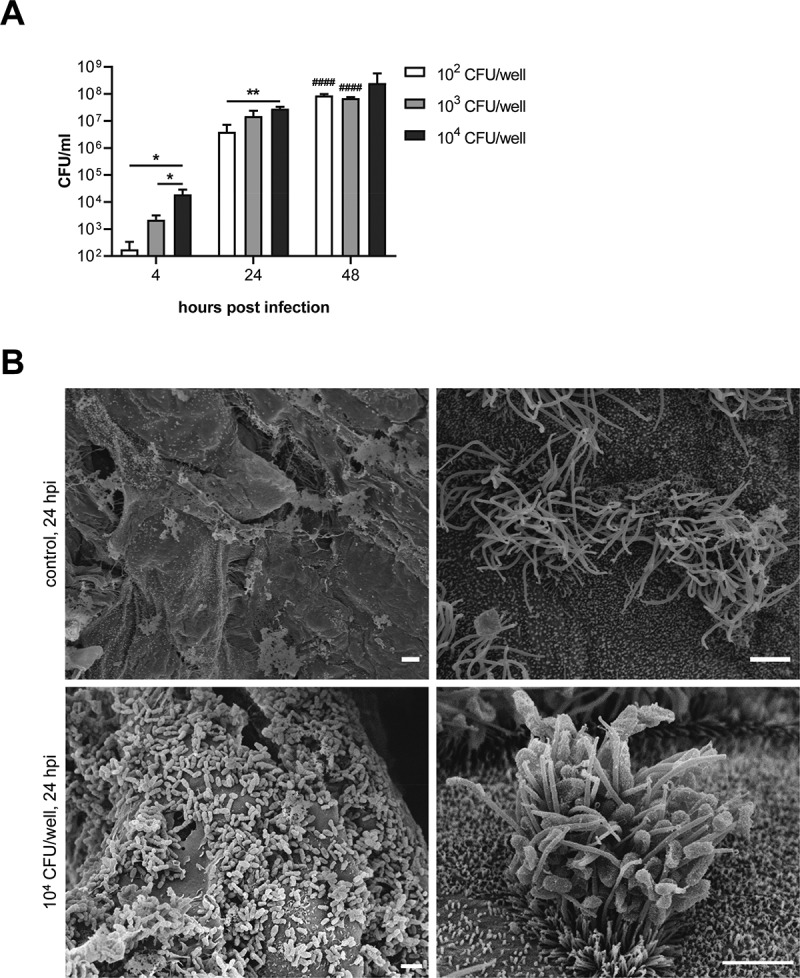
Colonization of PCLS by *B. bronchiseptica*. PCLS were infected with 10^2^–10^4^ CFU/well of *B. bronchiseptica* for up to 48 h. (A) To calculate the number of bacteria attached to the tissue, infected PCLS were homogenized at 4, 24, and 48 hpi and the lysate was plated on blood agar plates to determine CFU/ml. All experiments were repeated three times and means ± SD are shown. Significant differences between the infection doses are indicated by * *p* < 0.05 and ** *p* < 0.01; one-way ANOVA followed by Tukey *post-hoc* test. Significant differences between 4 hpi and 24 hpi or 48 hpi are indicated by ^####^ *p* < 0.0001; one-way ANOVA followed by Dunnett *post-hoc* test. (b) Field emission scanning electron microscopy of uninfected PCLS (control) and PCLS infected with 10^4^ CFU/well of *B. bronchiseptica* at 24 hpi. The images show *B. bronchiseptica* attached to the lung tissue (left image), in particular to the cilia (right image). Bars represent 2 μm

Our first objective was to establish defined conditions for the co-infection with *B. bronchiseptica* and *S. suis* in PCLS. Since *B. bronchiseptica* can cause reduction of ciliary activity of respiratory epithelial cells, as well as more severe mucosal damage, we first established conditions at which only ciliary activity was reduced without any further damage. For this, we infected the PCLS with low doses of *B. bronchiseptica* (10^2^–10^4^ CFU/well) for up to 48 h. Even the lowest infection dose of 10^2^ CFU/well reduced the ciliary activity at 24 hpi significantly, and 10^4^ CFU/well led to complete ciliostasis (Figure 1(a)). At 48 hpi, no beating of the cilia was observed in PCLS infected with any of the infection doses (Figure 1(a)). By measuring the release of LDH in the supernatant of infected PCLS, we evaluated the extent of cytotoxicity induced by *B. bronchiseptica*. Notably, we detected only low amounts of LDH in the supernatant and even the highest infection dose of 10^4^ CFU/well caused only a maximum of 10% LDH release at 48 hpi (Figure 1(b)). HE staining and immunofluorescence staining of PCLS infected with 10^4^ CFU/well confirmed these results, as almost no damage of the bronchiolar epithelium (loss of ciliated cells) was detectable at 24 hpi (Figure 1(c,d)). After 48 h, all infection doses led to a small number of detached ciliated cells (Figure S1, indicated by arrows), but no substantial loss of the bronchiolar epithelium was observed. Furthermore, immunofluorescence analysis showed that *B. bronchiseptica* adhered preferentially to the cilia (Figure 1(d)). Counting the number of PCLS-associated bacteria revealed a time- and dose-dependent increase of adherent and/or invasive bacteria until 24 hpi, but the recovered CFU at 48 hpi was independent of the initial infection dose (Figure 2(a)). FESEM confirmed the high colonization capacity of *B. bronchiseptica* to the lung tissue, in particular to the cilia (Figure 2(b)). Taken together, we established an infection dose of *B. bronchiseptica* (10^4^ CFU/well for 24 h), which is sufficient for bacterial colonization of the lung tissue and for a reproducible reduction of the ciliary activity without causing substantial damage to the bronchiolar epithelium.

### Prior infection with B. bronchiseptica promotes adherence and colonization of S. suis

**Figure 3.**
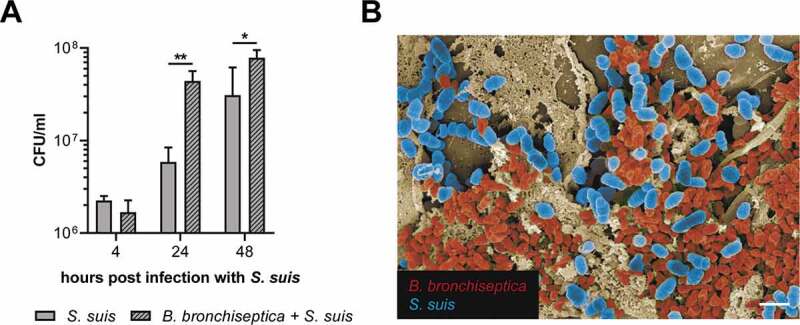
Colonization of PCLS by *B. bronchiseptica* and *S. suis*. PCLS were pre-infected with 10^4^ CFU/well of *B. bronchiseptica* for 24 h. Subsequently, PCLS were co-infected with 10^7^ CFU/well of *S. suis* serotype 2 wild-type strain 10 for up to 48 h. (A) To calculate the amount of *S. suis* attached to the tissue, mono- and co-infected PCLS were homogenized at 4, 24, and 48 hpi and the lysate was plated on Staph/Strep-agar plates to determine CFU/ml. The experiment was repeated at least three times and means ± SD are shown. Significant differences between mono- and co-infection are indicated by * *p* < 0.05 and ** *p* < 0.01; *t*-test. (B) Colorized FESEM of PCLS co-infected with *B. bronchiseptica* and *S. suis* for 48 h. This exemplary picture shows co-colonization of the lung tissue by *B. bronchiseptica* (red) and *S. suis* (blue). Bar represents 2 μm

**Figure 4.**
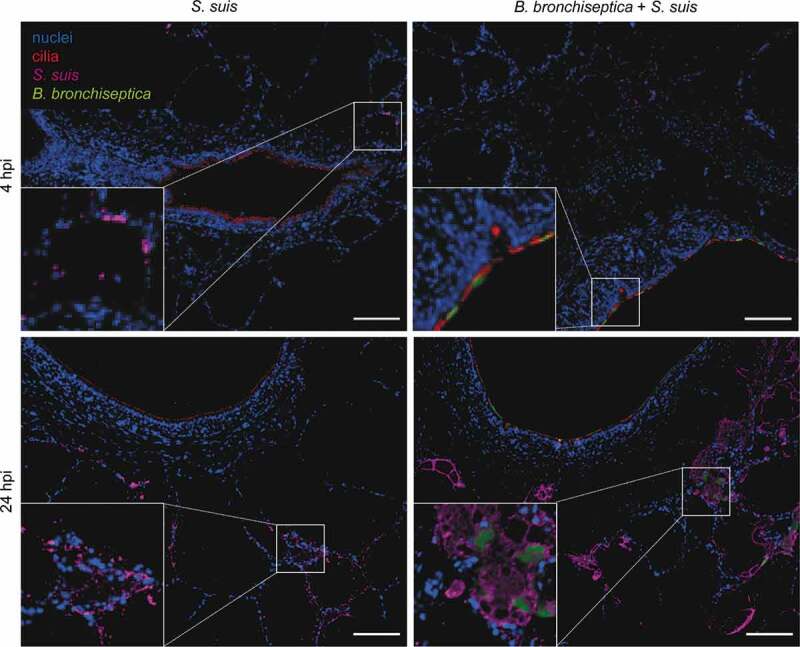
*B. bronchiseptica* and *S. suis* colonize different areas of PCLS. Immunofluorescence staining of mono- and co-infected PCLS at 4 and 24 hpi. *S. suis* is shown in magenta, *B. bronchiseptica* in green, cilia (β-tubulin) in red, and nuclei (DAPI) in blue. One exemplary picture is shown for each treatment. Bars represent 100 μm

Next, we investigated effects of prior infection with *B. bronchiseptica* on subsequent adherence and colonization of *S. suis*. PCLS were pre-infected with 10^4^ CFU/well of *B. bronchiseptica* for 24 h. We controlled the ciliary activity by light microscopy and confirmed a reduced motility due to infection with *B. bronchiseptica* (Figure S2) before PCLS were co-infected with 10^7^ CFU/well of *S. suis* for up to 48 h. Mono- and co-infected PCLS were homogenized to determine the number of tissue-associated *S. suis*. Interestingly, at 24 and at 48 hpi significantly more streptococci were counted in the lysates of PCLS pre-infected with *B. bronchiseptica* compared to PCLS mono-infected with *S. suis* (Figure 3(a)). Similar results were obtained by plating the supernatant of mono- and co-infected PCLS (Figure S3). FESEM of PCLS co-infected with *B. bronchiseptica* and *S. suis* for 48 h allowed us to visualize the co-colonization of the lung tissue by both pathogens, even though this situation was only rarely observed (Figure 3(b)). Immunofluorescence staining of mono- and co-infected PCLS showed an enhanced signal for streptococci in PCLS pre-infected with *B. bronchiseptica* at 24 hpi compared to the mono-infected sample (Figure 4). At 4 hpi no difference in the signal for *S. suis* was detectable between mono- and co-infected PCLS (Figure 4), confirming the plating results of the tissue lysates. Moreover, immunofluorescence analysis revealed that *B. bronchiseptica* bound preferentially to the cilia, whereas *S. suis* was mainly associated with the alveolar epithelium (Figure 4). Nevertheless, co-colonization of both pathogens was also observed sometimes (Figure 4). Taken together, we found that pre-infection of PCLS with *B. bronchiseptica* enhances the colonization capacity of *S. suis*. Notably, both pathogens seem to adhere to and colonize different areas of the tissue.

### Prior infection with B. bronchiseptica promotes cytotoxic effects of S. suis

**Figure 5.**
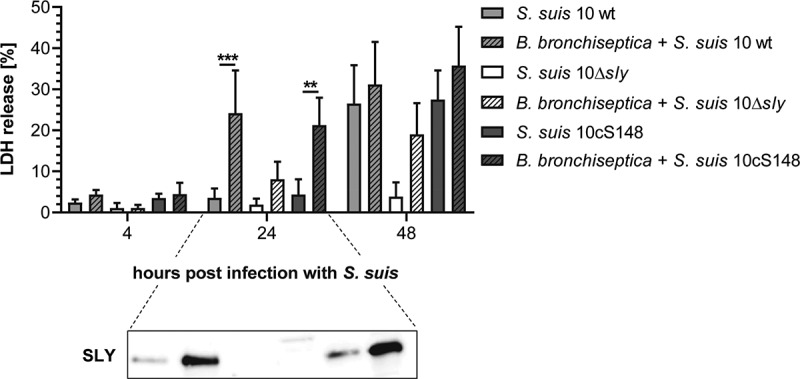
Cell damage in PCLS co-infected with *B. bronchiseptica* and *S. suis*. PCLS were pre-infected with 10^4^ CFU/well of *B. bronchiseptica* for 24 h. Subsequently, PCLS were co-infected with 10^7^ CFU/well of *S. suis* serotype 2 wild-type strain 10 (*S. suis* 10 wt), its isogenic SLY-deficient mutant strain (*S. suis* 10Δ*sly*), and the complemented SLY-mutant strain (*S. suis* 10cS148), respectively, for up to 48 h. Cytotoxicity was determined by measuring the release of LDH in the supernatant of mono- and co-infected PCLS. Results are expressed as percentage LDH release compared to PCLS lysed with 10% Triton® X 100. The experiment was repeated at least three times and means ± SD are shown. Significant differences between mono- and co-infection are indicated by ** *p* < 0.01 and *** *p* < 0.001; one-way ANOVA followed by Sidak *post-hoc* test. Below, immunoblot analysis of SLY-expression in the supernatant of mono- and co-infected PCLS at 24 hpi is shown

Next, we wanted to investigate whether the enhanced adherence and colonization of *S. suis* due to pre-infection with *B. bronchiseptica* resulted in higher cytotoxicity caused by *S. suis*. Moreover, we wanted to evaluate the role of SLY, the pore-forming toxin secreted by *S. suis*, which is considered a virulence-associated factor, in PCLS co-infected with *B. bronchiseptica* and *S. suis*. After pre-infection with *B. bronchiseptica* for 24 h, PCLS were co-infected with 10^7^ CFU/well of the virulent *S. suis* serotype 2 wild-type strain 10 (*S. suis* 10 wt), its isogenic SLY-deficient mutant (*S. suis* 10Δ*sly*), or the complemented SLY-mutant strain (*S. suis* 10cS148) for up to 48 h. We measured the release of LDH in the supernatant of PCLS either mono-infected with *S. suis* or co-infected with both pathogens to determine the cytotoxic effects caused by *S. suis*. We observed, that cytotoxicity induced by *S. suis* alone was strictly dependent on the presence of SLY, as almost no release of LDH was detected when PCLS were infected with *S. suis* 10Δ*sly*, whereas *S. suis* 10cS148 restored the phenotype of the *S. suis* 10 wt strain (Figure 5). Both, the SLY-deficient mutant strain and the complemented SLY-mutant strain showed similar colonization capacities compared to the wild-type strain (Figure S4). Interestingly, SLY-dependent release of LDH was significantly higher at 24 hpi in PCLS pre-infected with *B. bronchiseptica* compared to PCLS infected with *S. suis* alone (Figure 5). The extent of cytotoxicity in PCLS co-infected with *B. bronchiseptica* and *S. suis* 10Δ*sly* was higher compared to PCLS mono-infected with *S. suis* 10Δ*sly*, but lower than in PCLS co-infected with *B. bronchiseptica* and the SLY-positive strains (Figure 5). Immunoblot analysis of SLY expression in the supernatant of mono- and co-infected PCLS revealed a higher amount of SLY in the supernatant of PCLS pre-infected with *B. bronchiseptica* (Figure 5, inlay). Thus, we could show that the enhanced colonization capacity of *S. suis*, due to pre-infection with *B. bronchiseptica*, goes along with an increased amount of SLY in the supernatant of co-infected PCLS, resulting in higher cytotoxic effects caused by the toxin. In summary, these results show that *B. bronchiseptica* can promote infection of *S. suis* by enhancing its adherence and colonization, and, consequently, its cytotoxicity.

## Discussion

Respiratory diseases in pigs are often caused by a combination of different pathogens, whereby infection with one pathogen can pave the way for secondary pathogens [1]. *B. bronchiseptica* and *S. suis* are two major bacterial pathogens in pigs, contributing to the PRDC. *S. suis* is a pathobiont, which is considered to become invasive and to cause systemic infections only when other pathogens and/or certain environmental factors promote its translocation through the respiratory epithelium [4,33,34].

In a previous study of our group, reduction of the ciliary activity by swIAV subtype H3N2 promoted adherence of *S. suis* and its invasion into deeper tissues [19]. Like swIAV, *B. bronchiseptica* is known to contribute to secondary bacterial infections [5–7], probably by reduction of ciliary activity due to the TCT [21–24]. Thus, the objective of our study was to establish a co-infection model with *B. bronchiseptica* and *S. suis* in PCLS, to analyze the effects of a primary infection with *B. bronchiseptica* on subsequent infection with an opportunistic pathogen, such as *S. suis*. Using a relatively low dose of *B. bronchiseptica* allowed us to focus on the effects of reduced ciliary activity without affecting the integrity of the respiratory epithelial barrier. Higher doses might lead to destruction of the respiratory epithelium, as *B. bronchiseptica* is also known to cause extrusion of ciliated cells [35–37]. This would represent a completely different starting condition for the co-infection with an opportunistic pathogen. However, both scenarios are conceivable under natural conditions.

Next to the establishment of an *ex vivo* co-infection model with *B. bronchiseptica* and *S. suis*, we were interested in the role of SLY, a virulence-associated factor of *S. suis*, in such a co-infection scenario. We found that cytotoxicity induced by *S. suis* was strictly dependent on the presence of SLY, as almost no release of LDH was detectable when PCLS were mono-infected with the SLY-deficient mutant strain. This is in good agreement with previous studies performed with cell lines and primary cells of varying host and tissue origin [15,38] (reviewed in [18]). In PCLS co-infected with *B. bronchiseptica* and the SLY-deficient mutant strain, it becomes apparent that the cytotoxic effect of *B. bronchiseptica* is independent of SLY production. A similar effect was observed in porcine respiratory epithelial cells grown under air-liquid interface conditions when co-infected with swIAV and *S. suis* [38]. Strikingly, co-infection of PCLS with *B. bronchiseptica* and SLY-positive streptococci led to significant increase in cytotoxicity at 24 hpi compared to PCLS mono-infected with either of the strains, which seems not to be solely an additive effect. However, it seems plausible, that this effect is attributed to a higher number of streptococci in co-infected PCLS and, consequently, a higher concentration of SLY present in the supernatant of co-infected PCLS. Indeed, we demonstrated that pre-infection with *B. bronchiseptica* enhanced the colonization of PCLS by *S. suis*, resulting in a higher amount of SLY present in the supernatant of co-infected PCLS and in higher cytotoxic effects caused by SLY.

Whether pre-infection with *B. bronchiseptica* only promotes the adherence of *S. suis* (attachment to cells) or also its invasiveness (capacity to penetrate into deeper tissues para- and/or transcellularly) remains to be elucidated, as we could not distinguish between bacteria attached to the tissue and intracellular bacteria in this study. To address this aspect in future studies, transwell cell culture systems, such as air-liquid interface cultures with primary porcine respiratory epithelial cells [15,38], may be applied.

The precise mechanism of how *B. bronchiseptica* promotes the colonization of *S. suis* (and other pathogens) remains to be elucidated. It is plausible to assume that ciliostasis induced by *B. bronchiseptica* leads to impairment of the mucociliary clearance and thereby supports attachment of secondary bacteria to ciliated cells and/or invasion into deeper tissue [21]. Reduction of the ciliary activity might be attributed to the TCT [24], a virulence factor released by *B. bronchiseptica*. In contrast to *B. bronchiseptica*, infection with *S. suis* had no impact on the ciliary activity (Figure S2).

Damage to the bronchial/bronchiolar epithelium, i.e., the loss of ciliated cells, has the same effect of compromising the mucociliary clearance [39]. Moreover, loss of the uppermost cell layer might facilitate the attachment of secondary bacteria by exposing other receptors (adhesins) present on deeper cell layers of the bronchial/bronchiolar epithelium [39]. However, in our study, immunofluorescence analysis and histological examination did not reveal any substantial loss of ciliated cells in PCLS infected with 10^4^ CFU/well of *B. bronchiseptica*.

Another explanation for the enhanced adherence of *S. suis* might be the exploitation of adhesins secreted by *B. bronchiseptica* [40]. For the closely relative human pathogen *B. pertussis*, it was shown that pretreatment with filamentous hemagglutinin, an adhesin, which is also secreted by *B. bronchiseptica*, increased the adherence of *S. pneumoniae* and *Haemophilus influenzae* to ciliated cells [20]. In co-infection studies with *S. suis* and swIAV, interaction of the viral hemagglutinin with the bacterial capsular sialic acids was identified to promote adherence of *S. suis* [19,41,42]. Whether or not interactions between the filamentous hemagglutinin of *B. bronchiseptica* and the capsular sialic acids or other surface-associated structures of our *S. suis* strain occur, has to be investigated in future studies. However, a direct binding of *S. suis* to *B. bronchiseptica*, as it was reported for the binding of *S. suis* to swIAV-infected cells [19,38,41], is rather unlikely, as a direct interaction between both pathogens was not observed by FESEM. Moreover, immunofluorescence analysis showed that *B. bronchiseptica* and *S. suis* mainly attach to different areas of the lung tissue, which also means that they do not compete for binding sites in the respiratory tract. It might also be possible that *B. bronchiseptica* promotes streptococcal growth indirectly by providing nutrients, as it has been reported for influenza virus and *S. pneumoniae* [43].

In conclusion, we established an *ex vivo* co-infection model with *B. bronchiseptica* and *S. suis* in PCLS for analyses of interactions between both pathogens and primary host cells. In particular, the model is suitable to study effects of (primary) pathogen-induced ciliostasis on subsequent infection with a secondary pathogen. Our results showed that pre-infection with *B. bronchiseptica* promotes infection with *S. suis* by enhancing adherence and colonization of *S. suis*, which leads to an increased cytotoxicity caused by *S. suis*. Despite the contribution of *B. bronchiseptica* to *S. suis* infection, cytotoxicity of *S. suis* depends on the presence of the pore-forming toxin SLY. Hence, in co-infection of PCLS, the ciliostatic effect of *B. bronchiseptica* and the cytotoxic effect of SLY may act synergistically to impair cellular functions. In future studies, it will be interesting to address the mechanisms how *B. bronchiseptica* contributes to infection with secondary bacterial agents and to investigate the interactions of bacterial-bacterial co-infections of the porcine respiratory tract in more detail, as they represent a common economic problem in the pig industry. Finally, understanding of these interactions may well be translated to general pathogenicity concepts of respiratory co-infections in other host species, including humans.

## Supplementary Material

Supplemental Material

## References

[1] Brockmeier SL, Halbur PG, Thacker EL. Porcine respiratory disease complex. In: Brogden KA, editor. Polymicrobial diseases. Washington (DC): ASM Press; 2002:231–258. 21735561

[2] Saade G, Deblanc C, Bougon J, et al. Coinfections and their molecular consequences in the porcine respiratory tract. Vet Res. 2020;51(1):80. 32546263 10.1186/s13567-020-00807-8 PMC7296899

[3] Opriessnig T, Gimenez-Lirola LG, Halbur PG. Polymicrobial respiratory disease in pigs. Anim Health Res Rev. 2011;12(2):133–148. 22152290 10.1017/S1466252311000120

[4] Votsch D, Willenborg M, Weldearegay YB, et al. Streptococcus suis - The “Two Faces” of a pathobiont in the porcine respiratory tract. Front Microbiol. 2018;9:480. 29599763 10.3389/fmicb.2018.00480 PMC5862822

[5] Brockmeier SL. Prior infection with Bordetella bronchiseptica increases nasal colonization by Haemophilus parasuis in swine. Vet Microbiol. 2004;99(1):75–78. 15019114 10.1016/j.vetmic.2003.08.013

[6] Vecht U, Arends JP, van der Molen EJ, et al. Differences in virulence between two strains of Streptococcus suis type II after experimentally induced infection of newborn germ-free pigs. Am J Vet Res. 1989;50(7):1037–1043. 2774320

[7] Vecht U, Wisselink HJ, van Dijk JE, et al. Virulence of Streptococcus suis type 2 strains in newborn germfree pigs depends on phenotype. Infect Immun. 1992;60(2):550–556. 1730489 10.1128/iai.60.2.550-556.1992 PMC257663

[8] Brockmeier SL, Palmer MV, Bolin SR, et al. Effects of intranasal inoculation with Bordetella bronchiseptica, porcine reproductive and respiratory syndrome virus, or a combination of both organisms on subsequent infection with Pasteurella multocida in pigs. Am J Vet Res. 2001;62(4):521–525. 11327458 10.2460/ajvr.2001.62.521

[9] Brockmeier SL, Register KB, Nicholson TL, et al. Bordetellosis. In: Zimmermann JJ, et al., editor. Diseases of Swine. Hoboken, NJ: John Wiley & Sons, Inc; 2019:767-777.

[10] Palzer A, Ritzmann M, Wolf G, et al. Associations between pathogens in healthy pigs and pigs with pneumonia. Vet Rec. 2008;162(9):267–271. 18310558 10.1136/vr.162.9.267

[11] Cotter PA, Jones AM. Phosphorelay control of virulence gene expression in Bordetella. Trends Microbiol. 2003;11(8):367–373. 12915094 10.1016/s0966-842x(03)00156-2

[12] Sanford SE, Tilker ME. Streptococcus suis type II-associated diseases in swine: observations of a one-year study. J Am Vet Med Assoc. 1982;181(7):673–676. 7141961

[13] Gottschalk M, Xu J, Calzas C, et al. Streptococcus suis: a new emerging or an old neglected zoonotic pathogen? Future Microbiol. 2010;5(3):371–391. 20210549 10.2217/fmb.10.2

[14] Wertheim HF, Nghia H, Taylor W, et al. Streptococcus suis: an emerging human pathogen. Clin Infect Dis. 2009;48(5):617–625. 19191650 10.1086/596763

[15] Meng F, Wu N-H, Seitz M, et al. Efficient suilysin-mediated invasion and apoptosis in porcine respiratory epithelial cells after streptococcal infection under air-liquid interface conditions. Sci Rep. 2016;6(1):26748. 27229328 10.1038/srep26748 PMC4882623

[16] Seitz M, Baums CG, Neis C, et al. Subcytolytic effects of suilysin on interaction of Streptococcus suis with epithelial cells. Vet Microbiol. 2013;167(3–4):584–591. 24095145 10.1016/j.vetmic.2013.09.010

[17] Norton PM, Rolph C, Ward PN, et al. Epithelial invasion and cell lysis by virulent strains of Streptococcus suis is enhanced by the presence of suilysin. FEMS Immunol Med Microbiol. 1999;26(1):25–35. 10518040 10.1111/j.1574-695X.1999.tb01369.x

[18] Tenenbaum T, Asmat TM, Seitz M, et al. Biological activities of suilysin: role in Streptococcus suis pathogenesis. Future Microbiol. 2016;11:941–954. 27357518 10.2217/fmb-2016-0028

[19] Meng F, Wu NH, Nerlich A, et al. Dynamic virus-bacterium interactions in a porcine precision-cut lung slice coinfection model: swine influenza virus paves the way for streptococcus suis infection in a two-step process. Infect Immun. 2015;83(7):2806–2815. 25916988 10.1128/IAI.00171-15 PMC4468551

[20] Tuomanen E. Piracy of adhesins: attachment of superinfecting pathogens to respiratory cilia by secreted adhesins of Bordetella pertussis. Infect Immun. 1986;54(3):905–908. 2877952 10.1128/iai.54.3.905-908.1986 PMC260259

[21] Anderton TL, Maskell DJ, Preston A. Ciliostasis is a key early event during colonization of canine tracheal tissue by Bordetella bronchiseptica. Microbiology. 2004;150(Pt 9):2843–2855. 15347744 10.1099/mic.0.27283-0

[22] Bemis DA, Kennedy JR. An improved system for studying the effect of Bordetella bronchiseptica on the ciliary activity of canine tracheal epithelial cells. J Infect Dis. 1981;144(4):349–357. 7288215 10.1093/infdis/144.4.349

[23] Bemis DA, Wilson SA. Influence of potential virulence determinants on Bordetella bronchiseptica-induced ciliostasis. Infect Immun. 1985;50(1):35–42. 2864315 10.1128/iai.50.1.35-42.1985 PMC262131

[24] Heiss LN, Flak TA, Lancaster JR Jr., et al. Nitric oxide mediates Bordetella pertussis tracheal cytotoxin damage to the respiratory epithelium. Infect Agents Dis. 1993;2(4):173–177. 8173789

[25] Meng F, Punyadarsaniya D, Uhlenbruck S, et al. Replication characteristics of swine influenza viruses in precision-cut lung slices reflect the virulence properties of the viruses. Vet Res. 2013;44:110. 24225030 10.1186/1297-9716-44-110 PMC3840634

[26] Punyadarsaniya D, Liang C-H, Winter C, et al. Infection of differentiated porcine airway epithelial cells by influenza virus: differential susceptibility to infection by porcine and avian viruses. PLoS One. 2011;6(12):e28429. 22174804 10.1371/journal.pone.0028429 PMC3235120

[27] Goris K, Uhlenbruck S, Schwegmann-Wessels C, et al. Differential sensitivity of differentiated epithelial cells to respiratory viruses reveals different viral strategies of host infection. J Virol. 2009;83(4):1962–1968. 19052091 10.1128/JVI.01271-08 PMC2643795

[28] Paddenberg R, Mermer P, Goldenberg A, et al. Videomorphometric analysis of hypoxic pulmonary vasoconstriction of intra-pulmonary arteries using murine precision cut lung slices. J Vis Exp. 2014;(83):e50970. 24458260 10.3791/50970 PMC4089409

[29] Smith HE, Damman M, van der Velde J, et al. Identification and characterization of the cps locus of Streptococcus suis serotype 2: the capsule protects against phagocytosis and is an important virulence factor. Infect Immun. 1999;67(4):1750–1756. 10085014 10.1128/iai.67.4.1750-1756.1999 PMC96524

[30] Benga L, Fulde M, Neis C, et al. Polysaccharide capsule and suilysin contribute to extracellular survival of Streptococcus suis co-cultivated with primary porcine phagocytes. Vet Microbiol. 2008;132(1–2):211–219. 18565698 10.1016/j.vetmic.2008.05.005

[31] Mulisch M, Welsch U. Romeis - Mikroskopische Technik. 19th ed. Berlin, Germany: Springer Verlag; 2015

[32] Benga L, Goethe R, Rohde M, et al. Non-encapsulated strains reveal novel insights in invasion and survival of Streptococcus suis in epithelial cells. Cell Microbiol. 2004;6(9):867–881. 15272867 10.1111/j.1462-5822.2004.00409.x

[33] Lin X, Huang C, Shi J, et al. Investigation of pathogenesis of H1N1 influenza virus and swine streptococcus suis serotype 2 co-infection in pigs by microarray analysis. PLoS One. 2015;10(4):e0124086. 25906258 10.1371/journal.pone.0124086 PMC4407888

[34] Thanawongnuwech R, Brown GB, Halbur PG, et al. Pathogenesis of porcine reproductive and respiratory syndrome virus-induced increase in susceptibility to streptococcus suis infection. Vet Pathol. 2000;37(2):143–152. 10714643 10.1354/vp.37-2-143

[35] Matsuyama T, Takino T. Scanning electronmicroscopic studies of Bordetella bronchiseptica on the rabbit tracheal mucosa. J Med Microbiol. 1980;13(1):159–161. 7359573 10.1099/00222615-13-1-159

[36] Sekiya K, Futaesaku Y, Nakase Y. Electron microscopic observations on ciliated epithelium of tracheal organ cultures infected with Bordetella bronchiseptica. Microbiol Immunol. 1989;33(2):111–121. 2716544 10.1111/j.1348-0421.1989.tb01503.x

[37] Sekiya K, Futaesaku Y, Nakase Y. Electron microscopic observations on tracheal epithelia of mice infected with Bordetella bronchiseptica. Microbiol Immunol. 1988;32(5):461–472. 3173144 10.1111/j.1348-0421.1988.tb01406.x

[38] Meng F, Tong J, Votsch D, et al. Viral coinfection replaces effects of suilysin on streptococcus suis adherence to and invasion of respiratory epithelial cells grown under air-liquid interface conditions. Infect Immun. 2019;87(8). 10.1128/IAI.00350-19 PMC6652749 31138613

[39] Wu NH, Yang W, Beineke A, et al. The differentiated airway epithelium infected by influenza viruses maintains the barrier function despite a dramatic loss of ciliated cells. Sci Rep. 2016;6:39668. 28004801 10.1038/srep39668 PMC5177954

[40] Nicholson TL, Brockmeier SL, Loving CL. Contribution of Bordetella bronchiseptica filamentous hemagglutinin and pertactin to respiratory disease in swine. Infect Immun. 2009;77(5):2136–2146. 19237531 10.1128/IAI.01379-08 PMC2681739

[41] Wu NH, Meng F, Seitz M, et al. Sialic acid-dependent interactions between influenza viruses and Streptococcus suis affect the infection of porcine tracheal cells. J Gen Virol. 2015;96(9):2557–2568. 26297001 10.1099/jgv.0.000223

[42] Wang Y, Gagnon CA, Savard C, et al. Capsular sialic acid of Streptococcus suis serotype 2 binds to swine influenza virus and enhances bacterial interactions with virus-infected tracheal epithelial cells. Infect Immun. 2013;81(12):4498–4508. 24082069 10.1128/IAI.00818-13 PMC3837972

[43] Siegel SJ, Roche AM, Weiser JN. Influenza promotes pneumococcal growth during coinfection by providing host sialylated substrates as a nutrient source. Cell Host Microbe. 2014;16(1):55–67. 25011108 10.1016/j.chom.2014.06.005 PMC4096718

