# Supplementary material for: Bordetella bronchiseptica promotes adherence, colonization, and cytotoxicity of Streptococcus suis in a porcine precision-cut lung slice model
Source: Virulence. 2020 Dec 29;12(1):84–95. doi: 10.1080/21505594.2020.1858604 (PMC7781633; doi:10.1080/21505594.2020.1858604)
Supplement: Supplemental Material [file KVIR_A_1858604_SM4455.docx]

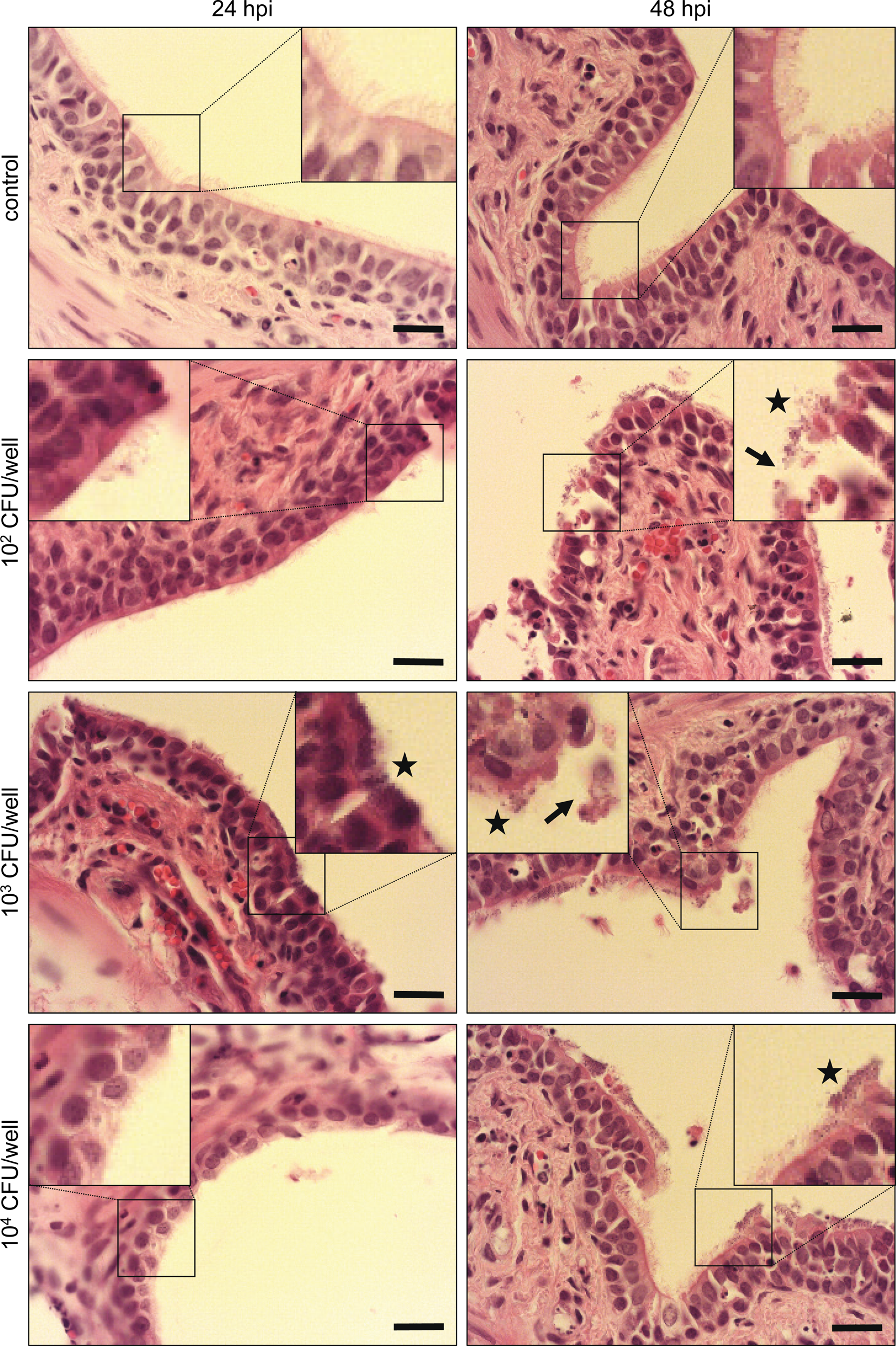


**Figure S1. Impact of *B. bronchiseptica* on PCLS.** HE staining of PCLS infected with 10^2^‑10^4^ CFU/well of *B. bronchiseptica* at 24 and 48 hpi. Damage of the bronchial epithelium (loss of cilia, disruption of epithelium) is indicated by arrows and accumulation of bacteria is indicated by asterisks. Bars represent 20 µm.


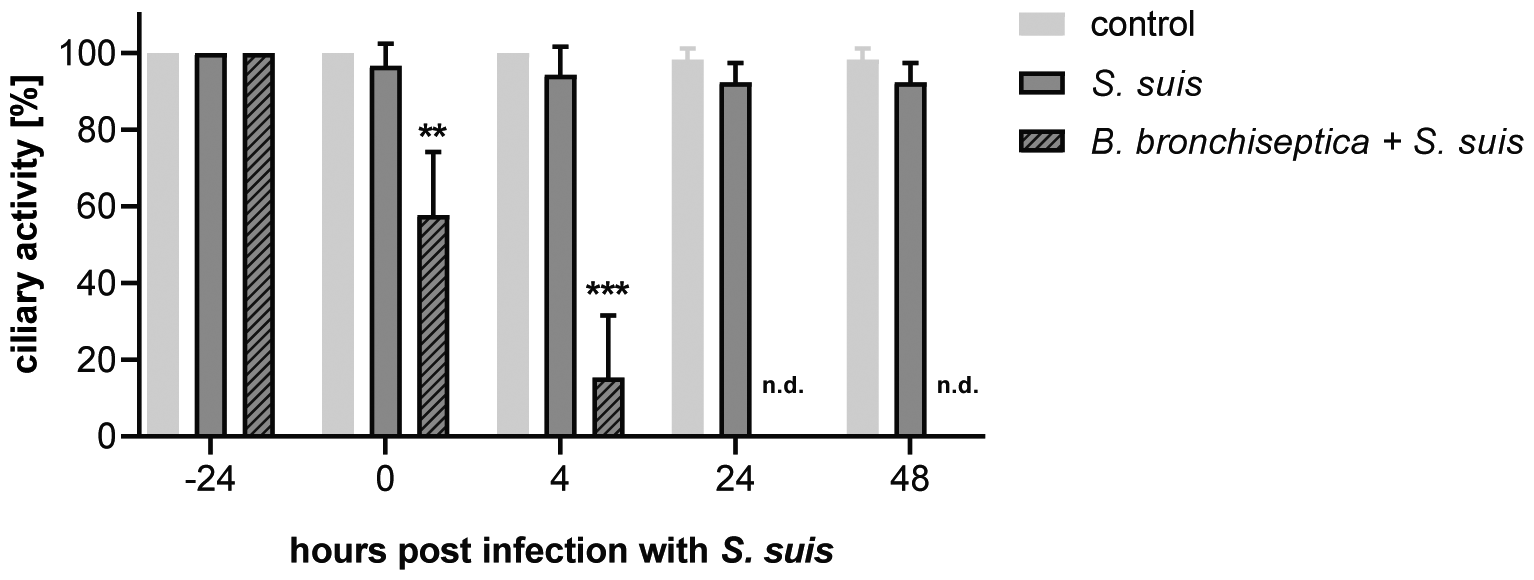


**Figure S2. Ciliary activity of PCLS co-infected with *B. bronchiseptica* and *S. suis*.** PCLS were pre-infected with 10^4^ CFU/well of *B. bronchiseptica* for 24 h. Subsequently, PCLS were co-infected with 10^7^ CFU/well of *S. suis* serotype 2 wild-type strain 10 for up to 48 h. Ciliary activity of uninfected (control) and infected PCLS was monitored at indicated time points by estimating the ciliary beating using light microscopy. Results are expressed as percentage ciliary activity compared to the ciliary activity before infection (set as 100%). Significant differences between uninfected and infected PCLS are indicated by ** *p* < 0.01 and *** *p* < 0.001; one-way ANOVA followed by Dunnett *post-hoc* test. Ciliary activity was not detectable (n.d.).


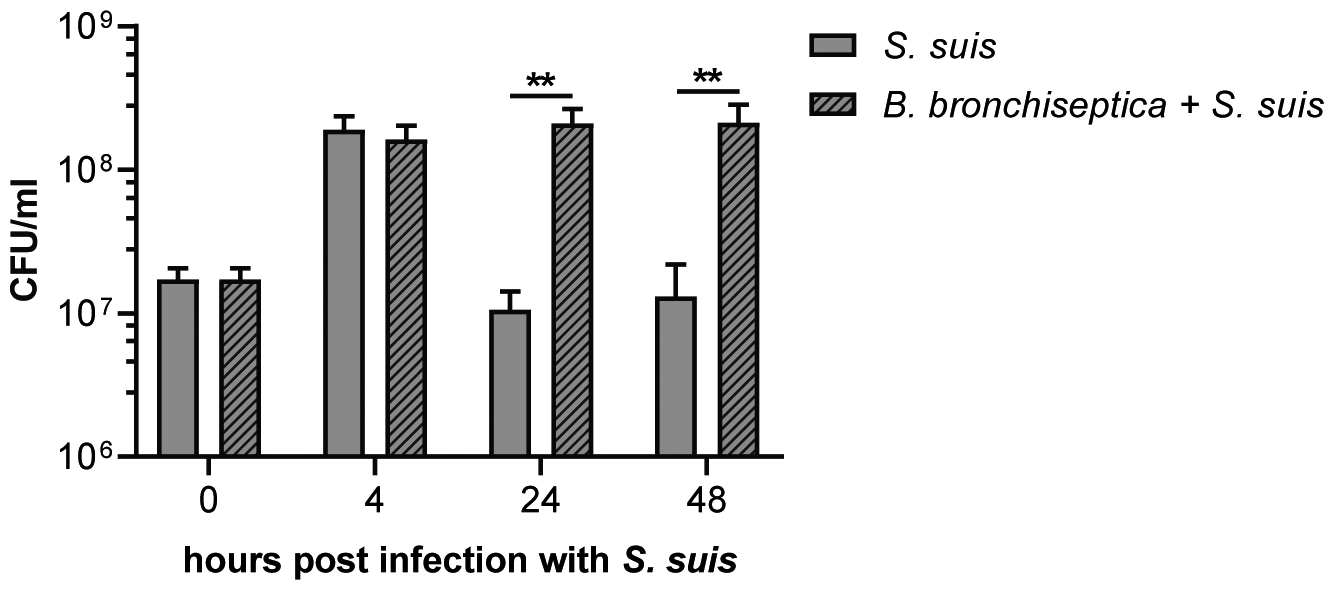


**Figure S3. Growth of *S. suis* in the supernatant of PCLS.** PCLS were infected with 10^4^ CFU/well of *B. bronchiseptica* for 24 h. Subsequently, PCLS were infected with 10^7^ CFU/well of *S. suis* serotype 2 wild-type strain 10 for up to 48 h. Replication of *S. suis* in the supernatant of infected PCLS was determined by serial dilution and replicate plating of the supernatant on Staph/strep-agar plates at indicated time points. Results are expressed as CFU/ml. The experiment was repeated at least three times and means ± SD are shown. Significant differences between mono- and co-infection are indicated by ** *p* < 0.01; *t*-test.


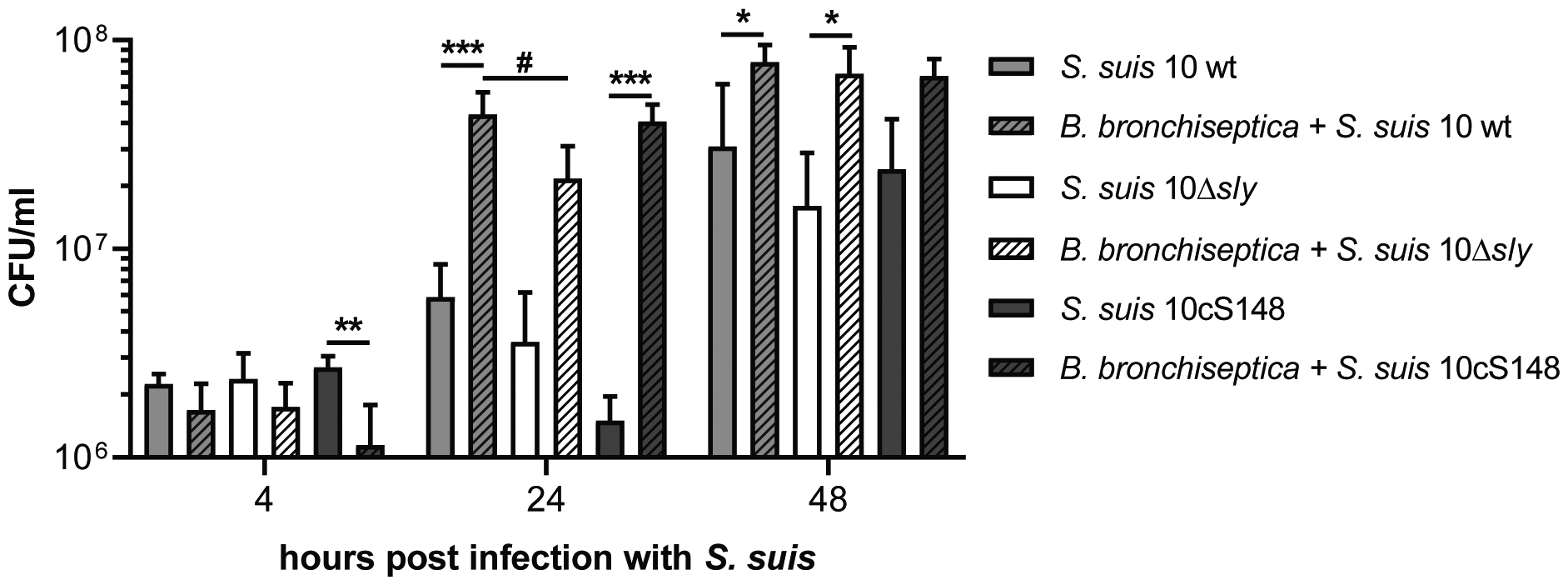


**Figure S4. Colonization of PCLS by *S. suis*.** PCLS were pre-infected with 10^4^ CFU/well of *B. bronchiseptica* for 24 h. Subsequently, PCLS were co-infected with 10^7^ CFU/well of *S. suis* serotype 2 wild-type strain 10 (*S. suis* 10 wt), its isogenic SLY-deficient mutant strain (*S. suis* 10Δ*sly*), and the complemented SLY-mutant strain (*S. suis* 10cS148), for up to 48 h. To calculate the amount of *S. suis* attached to the tissue, mono- and co-infected PCLS were homogenized at 4, 24, and 48 hpi and the lysate was plated on Staph/Strep-agar plates to determine CFU/ml. The experiment was repeated at least three times and means ± SD are shown. Significant differences between mono- and co-infection are indicated by * *p* < 0.05, ** *p* < 0.01, and *** *p* < 0.001, significant differences between the strains are indicated by ^#^ *p* < 0.05; one way-ANOVA followed by Tukey *post-hoc* test.
